# Changes in *CEBPB* expression in circulating leukocytes following eccentric elbow-flexion exercise

**DOI:** 10.1007/s12576-014-0350-7

**Published:** 2014-11-13

**Authors:** Jamie Blackwell, Lorna W. Harries, Luke C. Pilling, Luigi Ferrucci, Andrew Jones, David Melzer

**Affiliations:** 1 Sport and Health Sciences, College of Life and Environmental Sciences, University of Exeter, St. Luke’s Campus, Exeter, UK; 2 Institute of Biomedical and Clinical Sciences, University of Exeter Medical School, University of Exeter, Exeter, Devon UK; 3 Epidemiology and Public Health, University of Exeter Medical School, University of Exeter, Barrack Road, Exeter, Devon EX2 5DW UK; 4 Longitudinal Studies Section, Clinical Research Branch, National Institute on Aging, NIH, Baltimore, MD USA

**Keywords:** Muscle strength, Inflammation, *CEBPB*, Macrophage, Muscle damage, Muscle repair

## Abstract

In mouse models, CCAAT enhancer-binding protein beta (*CEBPB)* is necessary for M2 macrophage-mediated regeneration after muscle injury. In humans, *CEBPB* expression in blood was strongly associated with muscle strength. In this study we aimed to test whether *CEBPB* expression in blood in people is increased 2 days after exercise designed to induce muscle damage and subsequent repair. Sixteen healthy male volunteers undertook elbow flexor exercises designed to induce acute muscle micro-damage. Peripheral blood samples were collected at baseline and days 1, 2, 4 and 7 following exercise. Expression of *CEBPB* and related genes were analysed by qRT-PCR. Extent of muscle damage was determined by decline in maximal voluntary isometric torque and by plasma creatine kinase activity. Nine subjects had peak (day 4) creatine kinase activity exceeding 10,000 U/l. In this subgroup, *CEBPB* expression was elevated from baseline to 2 days post exercise (paired-samples *t*_(1,8)_ = 3.72, *p* = 0.006). Related expression and selected cytokine changes after exercise did not reach significance. Muscle-damaging exercise in humans can be followed by induction of *CEBPB* transcript expression in peripheral blood. Associations between *CEBPB* expression in blood and muscle strength may be consistent with the *CEBPB*-dependent muscle repair process.

**Electronic supplementary material:**

The online version of this article (doi:10.1007/s12576-014-0350-7) contains supplementary material, which is available to authorized users.

## Introduction

After muscle injury or change of use, repair starts with an initial acute inflammatory response (typically from 0 to 48 h) followed by a repair and regeneration phase, typically lasting from 48 h to 10 days [1]. CCAAT/enhancer-binding protein-beta (C/EBP-β, encoded for by *CEBPB* mRNA) is a pleiotropic transcription factor that regulates several immune cell functions. C/EBP-β has been shown to be central to post-injury muscle repair since *CEBPB* expression is markedly increased during macrophage differentiation towards an anti-inflammatory (M2) ‘repair’ phenotype that governs the integration of newly formed myoblasts into regenerating muscle tissue [2].

In a murine model of repair after a muscle injury, deletion of cAMP response element-binding protein (CREB) binding sites in the C/EBP-β promoter (thereby reducing *CEBPB* expression) in macrophages prevented the transition to an M2 regrowth and repair phenotype and inhibited muscle regeneration, resulting in fibrosis and muscle fibre loss, compromising muscle function [3].

Following injury, skeletal muscle undergoes a program of regeneration. Satellite progenitor cells proliferate as myoblasts and fuse into myotubes at the site of damage, where they differentiate into fully regenerated muscle fibres [4]. This sequence is mediated by a cascade of myogenic regulatory factors, which are subject to signalling from resident or infiltrating macrophages, and systemic factors originating in T lymphocytes. The proliferative phase of myoblasts is promoted by pro-inflammatory (M1) macrophages. Subsequently, alternatively activated M2 macrophages stimulate myotube differentiation [5]. M2 signal induction is mediated by Th_2_ cytokines [6].

We have previously demonstrated that the expression of the *CEBPB* gene in blood was the most strongly transcript associated with grip strength in 698 mostly older people in the InCHIANTI study in a transcriptome-wide analysis of human circulating blood leukocytes [7]. A potential mechanism explaining the strength-blood *CEBPB* association may be the role of C/EBP-β in muscle regeneration following lifelong muscle stress and injury [3]. Contraction-induced damage to muscle is common in everyday life, especially during lengthening contractions, and decreased repair after such damage has been linked to muscle strength, particularly in old age [8].

In the current study, we aimed to establish whether exercise-induced muscle damage (EIMD) in humans results in increases in *CEBPB* expression in blood, consistent with *CEBPB*’s role in post-injury muscle repair, which typically shows sharply increasing numbers of M2 macrophages at the injury site by 48 h after injury [1]. We also tested whether blood *CEBPB* and related expression and plasma cytokines exhibit changes consistent with the muscle regeneration program in human volunteers following EIMD. Here we present evidence that *CEBPB* expression is altered with EIMD, and *CEBPB* expression is associated with changes in markers characteristic of inflammatory polarisation.

## Methods

Sixteen healthy and physically active males [age, $\bar{x}$ (±SD) = 43.1 (18.5) years] undertook three sets of eccentric loading, 80 % 1RM [$\bar{x}$ (±SD) = 25.4 (5.1) kg, each set to task failure, separated by 2 min recovery] of the elbow flexors. Eccentric elbow flexion exercise is known to induce structural damage in humans [9]. The exercise protocol we used is consistent with inflammation in the biceps brachii and brachialis muscles [10]; maximal voluntary contraction (MVC) was assessed at 60° of flexion to allow potentially a greater contribution of damaged brachialis muscle than at 90°. MVC was assessed (peak torque, 5 × 3 s isometric contractions/3 s rest) prior to exercise and during recovery on an isokinetic dynamometer (Biodex System 3, Biodex Medical Systems, NY, USA). This project was approved by the ethics committee of the Department of Sport and Health Sciences, University of Exeter. Informed consent forms were signed by participants. Exclusion criteria included hypertension, injury, illness and medications related to inflammation.

Six millilitres of venous blood was collected into a lithium heparin Vacutainer (Becton-Dickinson, USA) and 2.5 ml into a PAXGene blood tube (PreAnalytix GmbH, Hombrechtikon, Switzerland) prior to and 1, 2, 4 and 7 days post exercise. These time points were chosen to coincide with the M1/M2 phenotype muscle regeneration model described by Tidball and Villalta [1]. The lithium heparin tube was centrifuged for 10 min at 4,000 RPM; the supernatant was harvested and analysed immediately for creatine kinase (CK) activity. The PAXGene tubes were inverted ten times, incubated at room temperature for ~3 h and stored without separation at −80 °C.

Creatine kinase activity was measured at 37 °C using the colorimetric technique described by Szasz [11]. Absorbance was recorded at λ 340 nm on a Jenway 6300 spectrophotometer (Bibby Scientific limited, Staffordshire, UK) and enzymatic activity quantified using a linear regression equation derived from a 2-point calibration of known standards.

Th1 and Th2 cytokines were quantified in plasma by electrochemiluminescence multiplex assay (Th1/Th2 10-plex, Meso Scale Discovery, Rockville, MD, USA). The panel of cytokines measured IFN-γ, IL-1β, IL-10, IL-12 p70, IL-13, IL-2, IL-4, IL-5, IL-8 and TNF-α.

For RNA extraction the PaxGene blood RNA kit (QIAGEN Ltd., Crawley, UK) and QiaCube automated system were used. mRNA was reverse transcripted to complimentary DNA using Superscript VILO (Applied Biosystems, USA) and amplified. cDNA was loaded onto custom TaqMan low density array (TLDA) cards on the ABI Prism 7900HT platform (Life Technologies, Carlsbad, CA, USA). Genes included in the study were quantified relative to an endogenous control gene (*PPIA*). Normalisation to *PPIA* precludes the need to correct for leucocytosis and has been well validated for its use as an appropriate housekeeping gene [12]. Samples were amplified in triplicate over 55 cycles. The quantitative value obtained from TaqMan real-time RT-PCR is a threshold cycle (CT). The fold differences between time conditions were calculated from the relativised CT values (CT gene X-CT housekeeping gene, *PPIA*) according to the comparative CT method. Genes quantified included seven associated with the ‘classical’ inflammatory T1 phenotype and 7 (including *CEBPB*) with an ‘alternative’ anti-inflammatory T2 phenotype (Table S1).

Within-group means were compared by paired *t*-tests for differences at a priori time points relevant to expected outcomes, in particular the rise from baseline to the mean of the subsequent 2 days, during which time M2 polarisation is expected to be induced. Relationships between mRNA expression and circulating cytokine concentrations were explored by Pearson’s correlation coefficients. The relationships between blood transcript expression changes and markers of damage severity (loss of function and CK activity) were assessed using robust regression analysis. Statistical significance was accepted at *p* < 0.05.

## Results

**Fig. 1:**
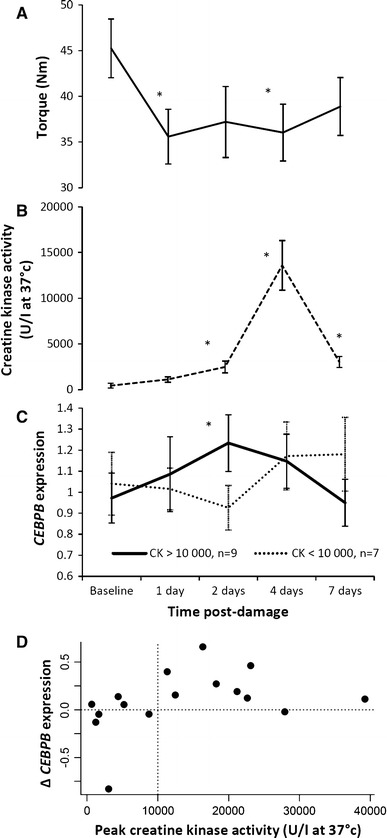
Time-course and relationship with *CEBPB* expression. Isometric strength (nm torque, ± SEM) decreases from baseline (**a**, *p < 0.05, *n* = 16) post-damage. Plasma creatine kinase activity (U/l, at 37 °C, ± SEM) increases from baseline (**b**, *p < 0.05, *n* = 16), peaking on day 4. Mean *CEBPB* blood transcript expression change (*CEBPB* quantification refers to *CEBPB* transcript expression relative to levels of the endogenous control gene *PPIA* and normalised to the mean *CEBPB* level over all samples, ± SEM) is higher at day 2 in *n* = 9 subjects with >10,000 peak plasma creatine kinase activity (**c**, *p = 0.006). **d** Shows the participants' change in *CEBPB expression* (day 2 minus baseline) and peak CK levels

Maximal voluntary contraction (MVC, 60° flexion) decreased from baseline and remained significantly depressed throughout the test period (pairwise repeated measures comparisons *p* < 0.05, Fig. 1a). Plasma CK activity (U/l, at 37 °C) increased from baseline (*p* < 0.05) with the greatest CK activity on day 4 (*p* < 0.05, Fig. 1b), although only 9 of the 16 subjects showed peak increases in CK exceeding 10,000 U/l. Peak CK activity for each participant was significantly associated with the maximum decrease in MVC (Spearman’s rho = −0.618, *p* = 0.013).

Overall *CEBPB* gene expression change after exercise (day 2, baseline) did not reach significance (*p* > 0.05). However in the subset whose peak CK activity exceeded 10,000 U/l, *CEBPB* expression did increase from baseline to 2 days post exercise (*t*_(1,8)_ = 3.72, *p* = 0.006, Fig. 1c, d). The same change in *CEBPB* expression was observed in a subset of ten subjects whose 24 h post exercise MVC decreased >17 % (*t*_(1,9)_ = 2.57, *p* = 0.03). The repeated measures of *CEBPB* expression over the 7 days did not significantly vary over time (ANOVA *p* > 0.05); only the day 2 change from baseline (in the high CK group) was significant (ANOVA *p* = 0.014). Supplementary Table 4 contains the *CEBPB* expression data at each time point and peak CK measure. There were no overall significant changes post exercise in the selected plasma cytokine concentration (Table S2) or other transcripts (Table S3), and none of these were significant in the high CK subgroup.

**Fig. 2:**
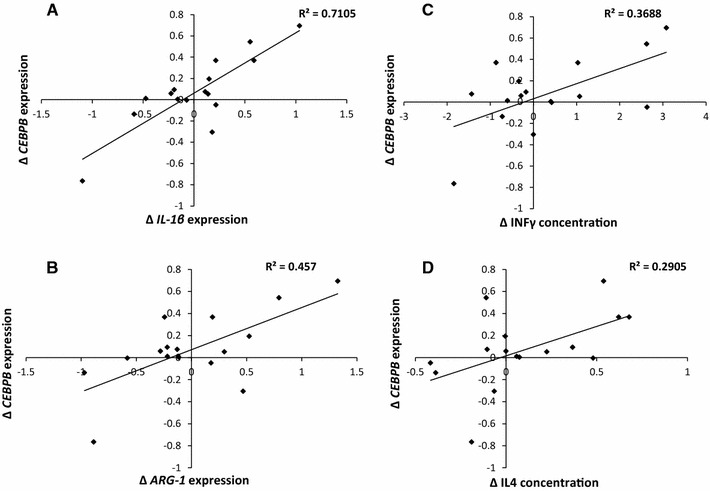
Associations of *CEBPB* expression to macrophage polarisation-associated markers. Following exercise-induced muscle damage, *CEBPB* blood transcript expression change shows positive correlations with *IL1β* (**a**, *p* = 4.14 × 10^−5^) and *ARG1* (**b**, *p* = 0.004) blood transcript expression change as well as change in circulating INF-γ (**c**, *p* = 0.013) and IL-4 concentration in plasma (**d**, *p* = 0.031) (∆*T* = [day 1 + day 2]/2 − baseline, *n* = 16)

Supporting the concept that *CEBPB* changes are related to T lymphocyte or macrophage differentiation, ∆ *CEBPB* expression correlated with ∆ *IL*-*1β* (*r* ^2^ = 0.701, *p* = 4.14 × 10^−5^, Fig. 2a), *ARG1* (*r* ^2^ = 0.457, *p* = 0.004, Fig. 2b) and *STAT1* (*r* ^2^ = 0.402, *p* = 0.008) expression. We also observed positive correlations between ∆*CEBPB* and ∆plasma INF-γ (*r* ^2^ = 0.369, *p* = 0.013, Fig. 2c) and IL4 concentration change (*r* ^2^ = 0.29, *p* = 0.031, Fig. 2c). ∆ = [day 1 + day 2]/2 − baseline. After negative changes in *CEBPB* expression had been excluded from the analysis, all the above associations remained significant except for interleukin 4.

## Discussion

In our previous ageing study, we showed that *CEBPB* expression in circulating leukocytes correlated with muscle strength in older people [7]. We hypothesised that this relationship may reflect the reported critical role *CEBPB* plays in leukocyte mediation of muscle regeneration following injury. We anticipated that a single bout of exercise-induced muscle damage would initiate a leukocyte-governed muscle regeneration response in which *CEBPB* expression would provide the essential switch between M1 and M2 inflammatory phenotypes. Thus, in a microcosm, we intended to create a model to assess the relevance of chronic circulating *CEBPB* expression that in future research could be applied to the study of age-related functional impairment. Here we present evidence that muscle-damaging exercise resulted in elevated blood *CEBPB* expression in volunteers exhibiting symptoms of more severe damage (elevated CK activity and decreased MVC). Although the degree of muscle damage was necessarily very small compared to the significant injuries explored in laboratory models, we found gene expression with similar direction and timing to those seen in models of the Th1/Th2 transition [1]. We also found suggestive evidence that *CEBPB* expression changes post exercise correlated with changes in Th1- and Th2-linked cytokine and transcript levels as hypothesised. These findings imply that in the exercise-induced muscle damage model, systemic inflammatory status may play a remote role in the governance of muscle remodelling, although more work is needed on this to provide definitive results.

Since we measured gene transcripts expressed in blood leukocytes, rather than in leukocytes resident in damaged muscle, we were challenged to justify our reasoning behind linking *CEBPB* expression to muscle remodelling. Much of the research into immunomodulatory effects on muscle regeneration has focused on the actions of resident macrophages. The differentiation of macrophages appears to occur in situ in damaged muscle [5] rather than prior to extravasation, and whether activated macrophages re-enter the circulation is unclear. Yet there may be a role of systemic T lymphocytes by either influencing myogenesis directly or remote macrophage phenotype induction [1]. Indeed, *CEBPB* is involved in the differentiation of T_H_0–T_H_1 and T_H_2 CD4+cells [13], and the molecular cascade that determines the inflammatory phenotype shares similarities with that of the macrophage. The relationships between *CEBPB* and Th1/Th2 markers may support this. Circulating IL4 may originate from regenerating muscle [14], known to stimulate *CEBPB* expression involved in M2 macrophage polarity. *CEBPB* expression is also influenced by IL1b [15], suggesting activation from pro-inflammatory leukocytes. We also report a relationship between *CEBPB* and *ARG1* expression change; M2 macrophages produce arginase 1, which competes with iNOS for l-arginine [6], thereby decreasing the M1 control of myogenic events [16]. In IL4-stimulated macrophages, *ARG*-*1* expression is dependent on C/EBP-β-mediated transcription [17]. These relationships provide some support that circulating *CEBPB* expression is related to Th1/Th2 inflammatory polarity. A role for peripheral factors in age-related muscle regeneration is supported by a murine study that infused serum from young mice into old, boosting the regeneration capacity [18].

Chronic low-grade systemic inflammation is well known to be related to decreases in muscle mass and functional capacity in longitudinal studies of aging [19–21]. Evidence of an anti-inflammatory antagonistic effect is sparse, although IL-10 is emerging as a key mediator of macrophage inflammatory status, associated with muscle regeneration [22]. As a transcription factor, *CEBPB* responds to environmental cues and elevates the production of IL-10, thus acting as an upstream mediator of the anti-inflammatory cascade, potentially ameliorating the negative consequences of inflammation. Induction of pro-inflammatory mediators of myoblast proliferation is essential for optimal muscle regeneration [23]; thus *CEBPB* may possibly be a target for pharmaceutical therapy to facilitate endogenous anti-inflammatory responses.

This study has several limitations, perhaps most importantly the relatively small sample size of 16, limiting statistical power. This small sample size likely explains why we did not detect an association between baseline muscle strength and baseline *CEBPB* expression, as previously reported in our analysis in 698 subjects [7]. The observed changes in MVC and CK suggest that structural damage and inflammation occurred in all volunteers, although evidence of inflammatory polarity in circulation was only observed in the most severely damaged individuals. The varying severity of damage and resulting disassociation from a universal inflammatory time course weakened potential main effects. Statistical power may be improved in future with the recruitment of a larger sample size or by employing laboratory techniques to improve the resolution of data. Creatine kinase is a widely used but imperfect measure of muscle injury, and follow-up studies could be strengthened by direct measures of injury, macrophage infiltration and repair. Changes in *CEBPB* expression from baseline to day 2 were variable between study subjects: in the group (*n* = 9) with CK changes above 10,000 U/l one individual showed essentially no change in *CEBPB* expression from baseline. Decreases in expression of *CEBPB* did occur in those with lower peak CK values and are likely to reflect the low baseline expression of this gene, against which small fluctuations in RT-PCR results appear to cause larger relative changes (a low signal-to-noise ratio).

We did not account for changes in leukocyte counts and subsets, but instead observed Th1/Th2 activity via circulatory factors and normalised rather than quantified gene expression. Measures of the abundance of specific cell subtypes (especially monocytes and macrophages) and gene expression within these cells could have provided more direct evidence linking *CEBPB* expression and macrophage activation. Future work might explore *CEBPB* and related expression after both a first and repeat bouts of exercise, as the latter should attenuate damage. We used *t*-tests in this analysis focussed, for example, on the change in *CEBPB* expression from baseline to day 2 rather than modelling the whole sequence of expression changes across all measures using a repeated measures ANOVA approach. This is because we expected a pattern of early increase in *CEBPB* expression by day 2 with a subsequent decline thereafter, given the dynamics of immune changes after muscle injury as summarised by Tidball and Villalta [1] and others. ANOVA tests of baseline and day 2 *CEBPB* expression gave similar results to *t*-tests, but repeat measures ANOVA for all *CEBPB* measures was not significant. In conclusion, *CEBPB* expression in blood was previously reported to be associated with muscle strength in humans and a suggested mechanism for this is that *CEBPB* expression in macrophages is necessary for repair after muscle injury. We therefore sought to test whether *CEBPB* expression in blood leukocytes is responsive to muscle injury inducing exercise. We have shown that in volunteers who likely sustained more severe muscle injury (i.e. those with CK levels >10,000 U/l) the expected increase in *CEBPB* expression in blood was present 48 h after exercise. Further study is needed to confirm the cellular origin of the *CEBPB* and related expression changes and to confirm the mechanistic relationship with downstream muscular regeneration events.

## Electronic supplementary material

Below is the link to the electronic supplementary material.

Supplementary material 1 (DOC 98 kb)
