# Supplementary material for: Changes in CEBPB expression in circulating leukocytes following eccentric elbow-flexion exercise
Source: J Physiol Sci. 2014 Nov 13;65(1):145–50. doi: 10.1007/s12576-014-0350-7 (PMC4276809; doi:10.1007/s12576-014-0350-7)
Supplement: Supplementary file 1 — Supplementary material 1 (DOC 98 kb) [file 12576_2014_350_MOESM1_ESM.doc]

**Supplementary information.**

**Tables**

Table S1. List of genes quantified by RT-PCR.

| **mRNA** | **Probe set ID** | **Protein** | **Function** | **Polarity** |
| --- | --- | --- | --- | --- |
| *ARG1* | Hs00163660_m1 | Arginase-1 | Competes with iNOS | M2 |
| *CCL2* | Hs00234140_m1 | Chemokine (C-C motif) ligand 2 | Promotion of M2 macrophages | M1 |
| *CEBPB* | Hs00942496_s1 | CCAAT-enhancer-binding protein beta, C/EBP-β | Transcription factor M2 polarisation | M2 |
| *IFNG* | Hs99999041_m1 | Interferon-γ | NFκβ1-induced M1 induction | M1 |
| *IL10* | Hs99999035_m1 | Interleukin-10 | Th2 produced cytokine promotes M2 induction. | M2 |
| *IL1B* | Hs01555413_m1 | Interleukin-1β | Acute-phase inflammatory cytokine | M1 |
| *IL4* | Hs00174122_m1 | interleukin-4 | Activator of M2 differentiation | M2 |
| *IL6* | Hs00174131_m1 | Interleukin-6 | Acute-phase inflammatory cytokine | M1 |
| *NFKB1* | Hs00231653_m1 | Nucleating factor κβ | Transcription factor M1 polarisation | M1 |
| *NOS2* | Hs01075529_m1 | Inducible nitric oxide synthase | NO mediation of inflammation | M1 |
| *PPIA* | Hs99999904_m1 | peptidylprolyl isomerase A | Housekeeping gene | Neutral |
| *STAT1* | Hs01014002_m1 | Signal transducer and activator of transcription 1 | Monocyte differentiation | M1 |
| *STAT6* | Hs00598618_m1 | Signal transducer and activator of transcription 6 | Transcription factor, activator of IL-4 | M2 |
| *TGFB3* | Hs00234245_m1 | Transforming growth factor β | Th2 inhibitor of Th1 induction | M2 |
| *TNF* | Hs00174128_m1 | Tumor necrosis factor-α | Pro-inflammatory cytokine | M1 |

Table S2. Cytokine values, mean (±standard error of the mean) pg/ml.

| Cytokine | baseline | 1 day | 2 day | 4 day | 7 day |
| --- | --- | --- | --- | --- | --- |
| INF | 1.51 (0.38) | 1.91 (0.66) | 1.73 (0.43) | 1.49 (0.31) | 1.80 (0.35) |
| IL-10 | 3.85 (0.73) | 4.15 (0.95) | 3.40 (0.74) | 3.84 (0.64) | 3.65 (0.42) |
| IL-12 | 0.73 (0.12) | 1.13 (0.17) | 1.06 (0.21) | 1.15 (0.20) | 1.13 (0.16) |
| IL-13 | 1.36 (0.36) | 2.77 (0.58) | 1.75 (0.44) | 1.50 (0.45) | 2.72 (0.54) |
| IL-1b | 0.13 (0.04) | 0.17 (0.04) | 0.10 (0.03) | 0.13 (0.04) | 0.39 (0.28) |
| IL-2 | 0.52 (0.21) | 0.33 (0.05) | 0.33 (0.06) | 0.50 (0.07) | 0.25 (0.05) |
| IL-4 | 0.38 (0.07) | 0.58 (0.07) | 0.40 (0.10) | 0.34 (0.09) | 0.27 (0.06) |
| IL-5 | 0.66 (0.13) | 0.75 (0.19) | 0.60 (0.08) | 0.78 (0.11) | 0.75 (0.14) |
| IL-8 | 5.42 (0.33) | 5.42 (0.28) | 4.98 (0.29) | 5.33 (0.45) | 5.44 (0.29) |
| TNF | 4.14 (0.28) | 4.18 (0.43) | 3.93 (0.34) | 4.24 (0.31) | 3.88 (0.33) |

Table S3. Median expression changes (fold change from baseline, normalised to *PPIA*) for whole leukoctye mRNA.

|  | Day 1 | Day 2 | Day 5 | Day 8 |
| --- | --- | --- | --- | --- |
| Δ-ARG1 | -0.10673 | -0.08279 | 0.098748 | 0.131909 |
| Δ-CCL2 | 0.126946 | 0.047975 | -0.07423 | -0.18606 |
| Δ-CEBPB | 0.076504 | 0.117974 | 0.153058 | 0.131998 |
| Δ-IFNG | -0.23747 | 0.036396 | -0.0905 | -0.34527 |
| Δ-IL10 | -0.158 | -0.0088 | 0.099666 | -0.02282 |
| ΔIL1B | 0.121334 | -0.04249 | 0.040802 | 0.082089 |
| Δ-IL4 | 0.162821 | 0.214585 | 0.105024 | 0.135871 |
| Δ-IL6 | 0.256955 | 0.356949 | 2.785703 | -2.28549 |
| Δ-NFKB1 | 0.084702 | -0.03689 | 0.00857 | 0.036142 |
| Δ-NOS2 | -0.0224 | -0.15965 | 0.264531 | 0.572353 |
| Δ-STAT1 | 0.039476 | -0.01191 | 0.159687 | -0.04803 |
| Δ-STAT6 | 0.066525 | -0.01228 | 0.139756 | 0.061345 |
| Δ-TGFB3 | 0.038145 | -0.12921 | 0.052389 | 0.122875 |
| Δ-TNF | 0.073087 | 0.006078 | 0.140433 | 0.073646 |

Table S4. *CEBPB* expression data at each time point in all sixteen participants, with peak CK data.

|  | ***CEBPB* expression** | | | | |  |
| --- | --- | --- | --- | --- | --- | --- |
| **Participant** | **Day 0** | **Day 1** | **Day 2** | **Day 4** | **Day 7** | **Peak CK** |
| 1 | 0.502498 | 0.929182 | 1.162850 | 1.276674 | 0.513515 | 16360 |
| 2 | 0.778861 | 0.677466 | 0.902581 | 0.930771 | 1.051028 | 22645 |
| 3 | 0.602853 | 0.731794 | 0.661173 | 1.249052 | 0.723988 | 670.3 |
| 4 | 0.968436 | 1.203861 | 0.837752 | 0.861607 | 1.318142 | 1243.5 |
| 5 | 1.490092 | 2.414989 | 1.953313 | 1.014931 | 1.631488 | 23115 |
| 6 | 1.734423 | 1.034894 | 0.904316 | 0.969756 | 0.821173 | 3060 |
| 7 | 1.164449 | 1.174576 | 1.303296 | 1.702107 | 1.511532 | 4411 |
| 8 | 1.308645 | 0.542474 | 1.464043 | 1.940456 | 0.899556 | 12470 |
| 9 | 0.755997 | 0.708862 | 0.810509 | 0.910203 | 0.878597 | 5227 |
| 10 | 1.297280 | 0.624950 | 1.695910 | 1.075735 | 0.569545 | 11330 |
| 11 | 0.404455 | 1.028263 | 0.516684 | 0.445062 | 0.947465 | 39240 |
| 12 | 1.185712 | 1.110196 | 1.163841 | 1.009066 | 0.927845 | 27930 |
| 13 | 0.813449 | 1.358973 | 1.004789 | 1.154863 | 1.309957 | 21200 |
| 14 | 0.970201 | 1.086847 | 1.242282 | 1.477348 | 0.696737 | 18240 |
| 15 | 0.632210 | 0.795439 | 0.585859 | 0.632570 | 0.899257 | 1665 |
| 16 | 1.425135 | 1.461496 | 1.379634 | 1.878650 | 2.111916 | 8745 |
